# Hidradenitis Suppurativa: Estimated Prevalence, Clinical Features, and Risk Factors in Riyadh, Saudi Arabia

**DOI:** 10.7759/cureus.23029

**Published:** 2022-03-10

**Authors:** Haifa Alsadhan, Abdulrahman I Alfawzan, Amirah Yaqoub, Alyah Almoneef, Mohammad Almohideb

**Affiliations:** 1 College of Medicine, King Saud Bin Abdulaziz University for Health Sciences, Riyadh, SAU; 2 Dermatology, King Abdulaziz Medical City Riyadh, Riyadh, SAU

**Keywords:** co-morbidities, risk factors, prevalence, clinical features, hidradenitis suppurativa

## Abstract

Background

Hidradenitis suppurativa (HS) is a chronic and recurrent inflammatory disease with a global prevalence of 1-4%, characterized by multiple painful nodules, abscesses, and fistulas that form scars in intertriginous regions (i.e., inguinal, axillary, mammary). HS is a complex and debilitating disease with a negative impact on quality of life. We aim to determine the prevalence, clinical features, risk factors, and comorbidities of HS.

Methodology

A retrospective, descriptive, cross-sectional study was conducted in King Abdulaziz Medical City from 2016 to 2020. Information of all confirmed cases of HS was extracted via computerized medical records. Data analysis was performed using SPSS version 24 (IBM Corp., Armonk, NY, USA). Categorical data were calculated based on frequency and percentage using the chi-square test to obtain p-values.

Results

Our initial search yielded 196 cases, of which 13 were excluded due to incomplete medical information. The prevalence of HS was 1.29%. The mean age was 27 years, with a male predominance. More than one-third of our patients were morbidly obese, and most of the patients were in Hurley stage 1 of both genders. The most commonly affected area was the axilla, followed by the inguinal area. The most common coexisting disease was diabetes mellitus type 2, followed by lipid disorders and acne.

Conclusions

This study documents the common demographic and clinical features of HS. It is a challenging disease in terms of detection and management, and it is critical to raise awareness among the public and physicians to minimize the devastating impact on HS patients.

## Introduction

Hidradenitis suppurativa (HS), a chronic and recurrent inflammatory disease, is characterized by multiple painful nodules, abscesses, and fistulas that form scars in sweat-bearing glands [1]. Due to numerous factors, including misdiagnosis, underdiagnosis, and delayed diagnosis, it is difficult to make an accurate estimate of its prevalence. The lowest reported prevalence is 0.00033% and the highest is 4.1% [2]. Although gender prevalence has been controversial in previous reports, HS has been shown to be more common among women with a ratio of 3.3:1 [3].

Associations with various risk factors of the cardiometabolic spectrum, such as diabetes mellitus, dyslipidemia, and hypertension, have been reported [4,5]. Smoking and obesity have been strongly associated with HS and are considered predominant exacerbating factors [6,7]. In addition, there is often a significant association with inflammatory bowel disease, most commonly Crohn’s disease [8].

Clinically relevant grading and assessment of disease severity are essential for the development of evidence-based treatments. Several grading systems have been introduced to evaluate the severity of HS, including Hurley staging, which uses clinical features, such as the presence of abscesses, scarring, and sinus tract formation, to classify HS patients into three distinct stages [9]. Although Hurley’s staging is considered the simplest, it is most commonly used in routine clinical practice to classify HS and is therefore essential for therapeutic decisions. However, because the classification is not quantitative, it cannot be used to measure the effectiveness of therapeutic interventions in clinical trials.

HS is a complex and debilitating disease that needs further research and information to be better understood. Unfortunately, diagnosis is long-delayed in many patients, possibly due to the lack of data regarding the risk factors and clinical features of HS in Saudi Arabia and Gulf countries combined with widespread misunderstanding, which leads to a significant burden for patients. Our aim in this study is to identify the prevalence, clinical features, and risk factors of HS in King Abdulaziz Medical City (KAMC), Riyadh, Saudi Arabia.

## Materials and methods

Study design, area, and setting

This is a retrospective, cross-sectional study conducted in KAMC, Riyadh, Saudi Arabia, from January 2016 to December 2020. We extracted data from electronic medical records of specialized dermatology clinics for all patients diagnosed with HS during the study period. We found 196 HS patients; however, we excluded 13 records due to incomplete medical information.

Data collection and study participants

A thorough medical record review was conducted in the dermatology department at KAMC after obtaining ethical approval from King Abdullah International Medical Research Center in Saudi Arabia. The diagnosis of HS, affected region, and Hurley stage were confirmed by dermatologists. Hurley stages are an indicator of HS severity and can be classified into three stages. Hurley stage 1 is defined as an abscess formation without sinus tracks or cicatrization, stage 2 is defined as a recurrent abscess with sinus tracks or cicatrization, and stage 3 is defined as diffuse area involvement with multiple interconnected tracks. In this study, Hurley stage 1 was considered mild, while Hurley stages 2 and 3 were considered severe. Data collected included demographic data such as age, sex, body mass index (BMI), family history of HS, and smoking status. We also collected data at the first and last visit for each patient with an average follow-up of five years. Data collected at the first and last visit included BMI, smoking status, and type of treatment. We considered that a patient underwent surgical treatment when there was a referral to surgery for surgical intervention. Comorbidities such as diabetes mellitus and hypertension were recorded during the first visit. Any comorbidity that occurred in less than four patients was included in the category of other comorbidities. We excluded patients diagnosed with HS outside KAMC. In addition, we excluded patients diagnosed with HS who never underwent treatment at KAMC.

Data analysis

Data entry was done using Google Forms for each patient. Data analysis was performed using SPSS version 24 (IBM Corp., Armonk, NY, USA). Categorical data were calculated based on frequency and percentage. The chi-square test was utilized to obtain p-values. When the data were non-parametric, Fisher’s exact test was utilized to obtain p-values. For changes in BMI after follow-up, a paired t-test was performed. A p-value equal to or less than 0.05 was considered significant.

## Results

The total number of referrals to dermatology during 2016-2020 was 15,221. The number of HS cases was 196. The calculated prevalence of HS was 1.29%.

Out of 183 HS patients, 101 were males. The mean age was 27.06 years (SD = 10.36). More than one-third of our patients were morbidly obese and most of them were in Hurley stage 1 in both genders. The most commonly affected area was the axilla, followed by the inguinal area. None of the patients were underweight, and the number of morbidly obese patients was higher than that of non-morbidly obese patients in all Hurley stages. In addition, demographic data compared with the Hurley stage did not yield a significant p-value. Additional demographic data can be found in Table 1.

**Table 1:** Demographic data. HS: hidradenitis suppurativa

Demographics, Number (%)	Hurley stage		P-value
	Mild	Severe	
Gender			
Male	55 (56.1)	46 (54.1)	0.451
Female	43 (43.9)	39 (45.9)	
Family history of HS			
No	93 (94.9)	81 (95.3)	0.865
Yes	5 (5.1)	4 (4.7)	
Body mass index			
Normal weight	16 (16.5)	7 (8.4)	0.245
Overweight	21 (21.6)	25 (30.1)	
Obese	28 (28.9)	20 (24.1)	
Morbid obese	32 (33)	31 (37.3)	

Table 2 shows the risk factors and the change of risk factors after an average follow-up of five years. Most smokers were males (N = 40, 93%), with a significant difference (p < 0.001). In addition, of the 43 smokers, only one male smoker had quit smoking after the first visit. The mean BMI of all patients showed no significant difference at the mean follow-up of five years. The mean BMI was 32 kg/m^2^ for males and 33 kg/m^2^ for females. In the first visit, 20 males started treatment, and 28 males had a surgical procedure, whereas during the first visit among females, eight started treatment, and 16 had a surgical procedure.

**Table 2:** Comparison of risk factors and types of treatments in males and females. BMI: body mass index

	Male	Female	P-value
Smoking first visit	40 (93)	3 (7)	<0.001
Smoking last visit	39 (92.8)	3 (7.1)	<0.001
Mean BMI first visit	32. 29	33.16	0.39
Mean BMI last visit	32.12	33.37	0.18
Adalimumab first visit	9 (52.9)	8 (47.1)	0.84
Adalimumab last visit	20 (19.8)	8 (9.8)	0.06
Surgical treatment first visit	28 (63.6)	16 (36.4)	0.2
Surgical treatment last visit	10 (62.5)	6 (37.5)	0.54

Table 3 shows the most commonly affected areas according to sex. The most common site was the axilla, affecting males more than females. The second most common region was the inguinal area with a significant difference between the genders (p = 0.05). Mammary involvement was observed in 20 female patients and one male patient (p < 0.001). In other regions, no significant difference was observed between gender and region affected by HS.

**Table 3:** Hidradenitis suppurativa-affected regions according to gender.

Region	Male	Female	Total	P-value
Axillary (%)	68 (56.3)	52 (43.7)	120	0.58
Inguinal/groin	49 (63.6)	28 (36.4)	77	0.05
Femoral	12 (57.1)	9 (42.9)	21	0.85
Perianal	9 (75)	3 (25)	12	0.15
Genital	9 (69.2)	4 (30.8)	13	0.29
Gluteal	22 (45.8)	26 (54.2)	48	0.13
Abdomen	7 (41.2)	10 (58.8)	17	0.22
Mammary	1 (4.8)	20 (95.2)	21	<0.001

**Figure 1:**
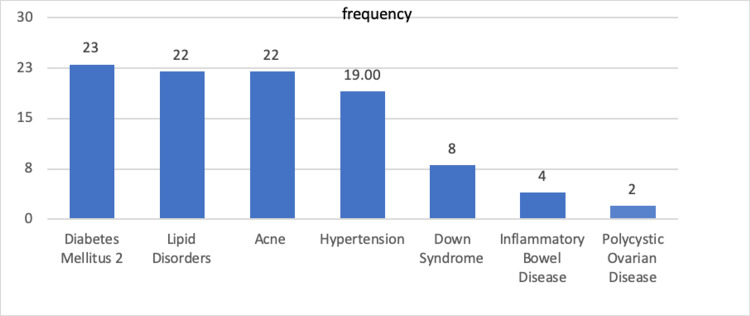
Frequency and percentage of common diseases in hidradenitis suppurativa patients.

Figure 1 shows the most common concomitant diseases associated with HS. The most common condition was diabetes mellitus type 2, followed by lipid disorders and acne, each affecting 12% of patients. Down syndrome was observed in eight patients.

## Discussion

A review of the literature on HS shows that reported prevalence in the general population varies from region to region. While data are inconsistent, most data range from 1% to 4% [1]. Our results fall within this range with a prevalence of 1.29%. Many studies have suggested that the prevalence may be higher than previously thought due to misdiagnosis or underdiagnosis. A Dutch study found that 72.6% of patients were female [10]. Observing the reported female-to-male ratio, it has been described that in otherwise healthy patients, the female-to-male ratio was 3.3:1 [3]. Interestingly, in contrast to the results of previous studies, our study showed a higher prevalence of males than females, 55.2% and 44.8%, respectively. This is thought to be due to limitations in reporting race-specific prevalence, as the ratio in African and Asian studies differs from that reported in the United States [2,11-14]. Another explanation is that women in our community do not seek medical advice because of shyness, which is supported by a previous study in Saudi Arabia [15].

Studies have shown that one-third of patients had a positive family history. These results support the notion that there is a genetic component in some HS patients, particularly mutations in g-secretase genes responsible for epidermal differentiation and proliferation [16,17]. However, in our study, only 4.9% of patients were found to have a positive family history. This could be due to underreporting of family history by physicians or patients’ lack of knowledge about their families’ medical history.

Our results showed that more than half of our patients had Hurley stage 1 (52.5%), and the majority of the patients were morbidly obese (34.4%). Obesity has been frequently cited as an independent risk factor associated with HS [18]. Several studies have suggested that higher BMI is associated with higher Hurley stages and worsening of disease severity. A common explanation is that obesity, although not a primary pathogenic factor, may exacerbate pre-existing HS by increasing skin shear force and possibly androgenic effects, leading to mechanical irritation, occlusion, and maceration [19-21]. However, in our study, no significant association was found between Hurley stage and BMI, although the majority of our patients were morbidly obese. However, it is important to note that the majority of the Saudi population are obese which could explain why patients with both mild and severe Hurley stage had the highest percentage of morbid obesity, and therefore, this could be masking the relation between BMI and HS disease severity [22].

An extensive literature addresses the risk factors associated with HS, such as obesity, smoking, and metabolic syndrome, which are significantly elevated compared to healthy controls, suggesting that HS is a systemic chronic inflammatory disease that is not limited to the skin [2,4,23]. Smoking is often cited as a recognized risk factor for both the development of HS and the severity of the disease as it can exacerbate the disease by acting as a pro-inflammatory stimulus or follicular occlusion promoter [24,25]. In a study involving 7,317 individuals in 13 regions of Saudi Arabia, the prevalence of smoking was found to be 32.5% in males and 3.9% in females [26]. Our results were largely consistent with the previously mentioned study and showed a drastically higher prevalence in males. It is noteworthy that the percentage of smokers did not decrease between the first and the last visit, as indicated in Table 2. We urge physicians to promote smoking cessation and point out how positively it can affect the course of disease in some patients.

An important point is which regions are affected by HS in both males and females. In females, the anterior region is typically affected, particularly the inguinal and mammary regions [3]. In contrast, HS has been observed in males in the posterior inguinal, gluteal, and atypical regions, such as the posterior thighs [3]. Our results show that the most common site in both males and females is the axilla, affecting more males (68%) than females (43.7%), which is consistent with a study from Turkey [27]. Another finding is that the second most common site was the inguinal region with a significant difference between the genders (p = 0.03). Males (63.6%) were more commonly affected than their female counterparts (36.4%). In addition, mammary involvement was observed in 20 female and one male patient with a significant difference (p < 0.001). These results are in direct agreement with previous findings [3,13,28]. However, no significance was found in relation to gender and regions affected by HS in other areas.

In our study, several comorbidities have been associated with HS. Ranking from the highest to the lowest association was diabetes mellitus type 2, followed by lipid disorders, acne, and, finally, hypertension. These findings are consistent with a meta-analysis study that found a significant association between HS and increased risk of cardiovascular and metabolic comorbidities [29]. In addition, another study found that metabolic syndrome was more prevalent in HS patients compared to controls (40% versus 13%), making metabolic and hormonal abnormalities a possible contributing factor to HS [30]. One study found that acne vulgaris was not significantly more common in patients with HS compared with normal controls, although 12% of HS patients in this study had acne vulgaris [31]. The least common diseases associated with HS were Down syndrome (DS), inflammatory bowel disease (IBD), and polycystic ovarian disease. A recent large population-based study found that patients with DS had a fivefold higher risk of developing HS than patients without DS [32]. A case series conducted in Saudi Arabia reported that 38% of DS patients also had HS, suggesting that there is a risk factor linking the two diseases [33]. In our study, DS had an overall prevalence of 4.4% among HS patients, suggesting that there might be underlying pathophysiology connecting the two diseases, as mentioned by Hamadah et al. [33]. The prevalence of IBD was 2.2%, which is inconsistent with a previous study that found IBD to be one of the most frequently reported comorbidities in patients with HS, raising the possibility of common pathogenesis [8].

Although we present one of the first local and regional papers determining prevalence, clinical features, risk factors, and comorbidities of HS, we encountered limitations. First, there might be some clinical features that were under-detected in this chart review which might result in the underreporting of many variables. In addition, the relatively low sample size compared to other studies in the literature could mask the extent of an association between HS and other variables. Lastly, this was a retrospective, single-center study that does not reflect the whole population of Saudi Arabia.

## Conclusions

This study provides a summary of epidemiology, clinical features, and comorbidities of 183 HS patients in a tertiary hospital in Riyadh, Saudi Arabia, that has not been studied previously. Statistically significant differences were reported between our variables with regard to gender and Hurley stage. We expect that our study contributes to the current knowledge of HS.

Due to the severity and chronic nature of the disease, it is critical to raise awareness among the public and physicians through educational campaigns for HS and to encourage patients to reduce modifiable risk factors, such as smoking cessation and weight loss, and advocate appropriate coping mechanisms. As for physicians, we would like to see methods of early detection and diagnosis that can lead to timely referral. To conclude, further prospective studies with a larger sample size are needed to establish more objective results.

## References

[1] Alikhan A, Lynch PJ, Eisen DB (2009). Hidradenitis suppurativa: a comprehensive review. J Am Acad Dermatol.

[2] Miller IM, McAndrew RJ, Hamzavi I (2016). Prevalence, risk factors, and comorbidities of hidradenitis suppurativa. Dermatol Clin.

[3] Canoui-Poitrine F, Revuz JE, Wolkenstein P (2009). Clinical characteristics of a series of 302 French patients with hidradenitis suppurativa, with an analysis of factors associated with disease severity. J Am Acad Dermatol.

[4] Revuz JE, Canoui-Poitrine F, Wolkenstein P (2008). Prevalence and factors associated with hidradenitis suppurativa: results from two case-control studies. J Am Acad Dermatol.

[5] Jiménez-Gallo D, de la Varga-Martínez R, Ossorio-García L, Collantes-Rodríguez C, Rodríguez C, Linares-Barrios M (2018). Effects of adalimumab on T-helper-17 lymphocyte- and neutrophil-related inflammatory serum markers in patients with moderate-to-severe hidradenitis suppurativa. Cytokine.

[6] Shahi V, Alikhan A, Vazquez BG, Weaver AL, Davis MD (2014). Prevalence of hidradenitis suppurativa: a population-based study in Olmsted County, Minnesota. Dermatology.

[7] Sartorius K, Emtestam L, Jemec GB, Lapins J (2009). Objective scoring of hidradenitis suppurativa reflecting the role of tobacco smoking and obesity. Br J Dermatol.

[8] Fimmel S, Zouboulis CC (2010). Comorbidities of hidradenitis suppurativa (acne inversa). Dermatoendocrinol.

[9] Hurley H (1989). Axillary hyperhidrosis, apocrine bromhidrosis, hidradenitis suppurative, and familial benign pemfigus: surgical approach. Dermatologic Surgery: Principles and Practice.

[10] Schrader AM, Deckers IE, van der Zee HH, Boer J, Prens EP (2014). Hidradenitis suppurativa: a retrospective study of 846 Dutch patients to identify factors associated with disease severity. J Am Acad Dermatol.

[11] Mebazaa A, Ben Hadid R, Cheikh Rouhou R (2009). Hidradenitis suppurativa: a disease with male predominance in Tunisia. Acta Dermatovenerol Alp Pannonica Adriat.

[12] Kurokawa I, Hayashi N (2015). Questionnaire surveillance of hidradenitis suppurativa in Japan. J Dermatol.

[13] Yang JH, Moon J, Kye YC (2018). Demographic and clinical features of hidradenitis suppurativa in Korea. J Dermatol.

[14] Lee DE, Clark AK, Shi VY (2017). Hidradenitis suppurativa: disease burden and etiology in skin of color. Dermatology.

[15] Shirah BH, Shirah HA (2017). The clinical pattern of axillary hidradenitis suppurativa among Saudi Arabians: mode of presentation and treatment challenges. J Cutan Aesthet Surg.

[16] Ingram JR (2016). The genetics of hidradenitis suppurativa. Dermatol Clin.

[17] Wang B, Yang W, Wen W (2010). Gamma-secretase gene mutations in familial acne inversa. Science.

[18] Shlyankevich J, Chen AJ, Kim GE, Kimball AB (2014). Hidradenitis suppurativa is a systemic disease with substantial comorbidity burden: a chart-verified case-control analysis. J Am Acad Dermatol.

[19] Theut Riis P, Saunte DM, Benhadou F (2018). Low and high body mass index in hidradenitis suppurativa patients-different subtypes?. J Eur Acad Dermatol Venereol.

[20] Slade DE, Powell BW, Mortimer PS (2003). Hidradenitis suppurativa: pathogenesis and management. Br J Plast Surg.

[21] Jemec GB (2003). Hidradenitis suppurativa. J Cutan Med Surg.

[22] Alghnam S, Alessy SA, Bosaad M (2021). The association between obesity and chronic conditions: results from a large electronic health records system in Saudi Arabia. Int J Environ Res Public Health.

[23] Miller IM, Ellervik C, Vinding GR, Zarchi K, Ibler KS, Knudsen KM, Jemec GB (2014). Association of metabolic syndrome and hidradenitis suppurativa. JAMA Dermatol.

[24] König A, Lehmann C, Rompel R, Happle R (1999). Cigarette smoking as a triggering factor of hidradenitis suppurativa. Dermatology.

[25] Vazquez BG, Alikhan A, Weaver AL, Wetter DA, Davis MD (2013). Incidence of hidradenitis suppurativa and associated factors: a population-based study of Olmsted County, Minnesota. J Invest Dermatol.

[26] Algabbani AM, Almubark R, Althumiri N, Alqahtani A, Bindhim N (2018). The prevalence of cigarette smoking in Saudi Arabia in 2018. Food Drug Regul Sci J.

[27] Yüksel M, Basım P (2020). Demographic and clinical features of hidradenitis suppurativa in Turkey. J Cutan Med Surg.

[28] Bianchi L, Caposiena Caro RD, Ganzetti G (2019). Sex-related differences of clinical features in hidradenitis suppurativa: analysis of an Italian-based cohort. Clin Exp Dermatol.

[29] Tzellos T, Zouboulis CC, Gulliver W, Cohen AD, Wolkenstein P, Jemec GB (2015). Cardiovascular disease risk factors in patients with hidradenitis suppurativa: a systematic review and meta-analysis of observational studies. Br J Dermatol.

[30] Sabat R, Chanwangpong A, Schneider-Burrus S (2012). Increased prevalence of metabolic syndrome in patients with acne inversa. PLoS One.

[31] Revuz J (2009). Hidradenitis suppurativa. J Eur Acad Dermatol Venereol.

[32] Garg A, Strunk A, Midura M, Papagermanos V, Pomerantz H (2018). Prevalence of hidradenitis suppurativa among patients with Down syndrome: a population-based cross-sectional analysis. Br J Dermatol.

[33] Hamadah I, Haider M, Chisti M, Binamer Y (2017). Hidradenitis suppurativa in Down syndrome: a case series. Pediatr Dermatol.

